# Assessing genome assembly quality using the LTR Assembly Index (LAI)

**DOI:** 10.1093/nar/gky730

**Published:** 2018-08-10

**Authors:** Shujun Ou, Jinfeng Chen, Ning Jiang

**Affiliations:** 1 Department of Horticulture, Michigan State University, East Lansing, MI 48824, USA; 2 Program in Ecology, Evolutionary Biology and Behavior, Michigan State University, East Lansing, MI 48824, USA; 3 Department of Plant Pathology and Microbiology, University of California, Riverside, CA 92507, USA

## Abstract

Assembling a plant genome is challenging due to the abundance of repetitive sequences, yet no standard is available to evaluate the assembly of repeat space. LTR retrotransposons (LTR-RTs) are the predominant interspersed repeat that is poorly assembled in draft genomes. Here, we propose a reference-free genome metric called LTR Assembly Index (LAI) that evaluates assembly continuity using LTR-RTs. After correcting for LTR-RT amplification dynamics, we show that LAI is independent of genome size, genomic LTR-RT content, and gene space evaluation metrics (i.e., BUSCO and CEGMA). By comparing genomic sequences produced by various sequencing techniques, we reveal the significant gain of assembly continuity by using long-read-based techniques over short-read-based methods. Moreover, LAI can facilitate iterative assembly improvement with assembler selection and identify low-quality genomic regions. To apply LAI, intact LTR-RTs and total LTR-RTs should contribute at least 0.1% and 5% to the genome size, respectively. The LAI program is freely available on GitHub: https://github.com/oushujun/LTR_retriever.

## INTRODUCTION

In the shotgun sequencing era, the assembly of a new genome is mostly reliant on computational algorithms. The performance of such algorithms, as well as read length, insertion size of sequencing libraries, read accuracy, and genome complexity, determine the accuracy and continuity of the genome assembly. Therefore, the quality of a genome assembly is hardly predictable. To evaluate the quality of a new assembly, several methods have been developed, which include contig size measurements, gene set completeness, misassembly evaluation, and synteny comparison. The contig N50, which is the shortest contig length at 50% of the total genome size, is widely used to estimate assembly continuity but could be misleading if short contigs are artificially concatenated (1). Similarly, scaffold N50 is a metric to reflect the continuity of a genome scaffold. Currently, the Benchmarking Universal Single-Copy Orthologs (BUSCO) and Core Eukaryotic Genes Mapping Approach (CEGMA) programs represent the ‘state-of-the-art’ methods for evaluation of gene space quality through the evaluation of presence or absence of numerous highly conserved orthologous genes (2,3). However, most newly assembled genomes (including draft genomes) are associated with high BUSCO and CEGMA scores, which is not sufficient to reveal the completeness of the whole genome. In contrast, the QUAST program compares genome assembler programs by estimating misassemblies in contig blocks (1), which is limited however by the availability of a reference genome.

Due to the repetitive nature of transposable elements (TEs), their assembly is notoriously difficult and unreliable (4). However, TEs are major components of most eukaryotic genomes and often interact with genes (5). To date, there are no established metrics available for the evaluation of repetitive sequence space (6). LTR retrotransposons (LTR-RTs) are interspersed repetitive elements that typically range from 4 to 20 Kb and dominate most plant genomes (4,5,7). Upon insertion, the long terminal repeat of the element is identical to each other, then base substitution will occur randomly and constantly on the LTR region based on the neutral theory, which can be used to infer the age of the insertion event (4). Deletion will also occur on LTR-RTs due to intra-element unequal homologous recombination and illegitimate recombination (8,9). Both substitution and deletion can alter the sequence and the structure of an intact LTR-RT, and eventually lead to degradation or removal (4,8). Intra-element recombination is thought to be the major process facilitating the removal of LTR sequence in genomes of rice (*Oryza sativa*) and *Arabidopsis lyrata* (8,9), resulting in the formation of solo LTRs that consist of only one of the LTR regions.

Identification of LTR elements using computer programs based on structural features is efficient (10,11), yet suffering from large numbers of false positives (4). Recently, the LTR_retriever software was developed for accurate *de novo* identification of intact LTR retrotransposons (4). This tool eliminates LTR false positives regardless of the input quality and has demonstrated ultrahigh sensitivity and accuracy with very low false discovery rate (4). While searching plant genomes for intact LTR elements, we observed that more intact elements could be identified from more completed genome assemblies compared with draft genomes. For example, there were 2,052 intact LTR-RTs retrieved from the well-assembled rice reference genome ‘Nipponbare’ (MSUv7 version), while only 239 intact LTR-RTs could be identified from the same genome sequenced using next-generation sequencing (NGS) technique (the assembly was obtained from (12)). Jiao *et al.* reported similar findings in the new maize (*Zea mays*) reference genome (v4) sequenced by PacBio long-read techniques (13). Al-Dous *et al.* also showed that short-read-based genome sequencing could resolve only a small fraction of long repeats like LTR-RTs in the date palm (*Phoenix dactylifera*) genome (14). These findings suggest that a more continuous genome assembly would result in more intact LTR elements being identified. Thus, the amount of identifiable intact LTR elements, in turn, can indicate the assembly quality of the intergenic and repetitive sequence space (Supplementary Figure S1).

## MATERIALS AND METHODS

### Collection of whole-genome sequences

A total of 103 genomes were collected and used in this study. These genomes contain at least 5% of LTR-RTs and were collected from Phytozome (41 genomes) (15), the National Center for Biotechnology Information (NCBI) (24 genomes), and a variety of specialty websites. Four versions of the *Solanum pennellii* genome sequenced using the Oxford Nanopore technique were obtained from Schmidt *et al.* (16). High-quality long-read based genomes were determined if contig N50 > 100 Kb, both BUSCO and CEGMA completeness > 80%, and either BUSCO or CEGMA completeness > 90%. For genomes decoded by multiple sequencing techniques, the dominating technique for contig construction was used to represent the genome. Details about these genomes were listed in Supplementary Table S1.

### Collection of high-quality BAC sequences

All plant BACs were obtained from the nucleotide database in NCBI with search criteria ‘BAC[All Fields] AND plants[filter]’. To filter out non-nuclear BACs, sequences with following keywords in the title were excluded: plastid, chloroplast, mitochondri, ribosomal, transposon, gene, plasmid, vector, virus, TINY, Micromonas, Podospora, Uncultured, Rdr1, Co-Gene, S-locus, Patent, zein, scaffold, and shotgun. Finished BACs with ‘complete sequence’ indicated in the title and sequence length ≥ 20 Kb were retained. For draft BACs with less than 10 gaps, the sequence pieces ≥ 20 Kb were also retained. BAC sequences of the same species were put together as one sample. Samples that were < 3 Mb in size or contained less than 5% of LTR sequences were not used in the analyses. The *Carica papaya* sample was removed due to the low abundance of intact LTR-RT (only 0.3% of the sample size). Finally, a total of 14,826 high-quality BAC sequences derived from 21 plant species were retained for subsequent analysis.

### Whole-genome forward simulation

To simulate evolution of genomes forward in time, a custom Perl script ‘simulate_mutation.pl’ was used to introduce random mutations to the genome. The script is available in the package of LTR_retriever. Percentage of mutations (‘-u’) ranging from 0.1% to 9% which is equivalent to evolution times of 0.04 to 3.46 million years (MY, μ = 1.3 × 10^−8^ per bp per year) were introduced to the original genome for the simulation of genomes. Simulated genomes were treated as new species with LTR-RT outbreaks that could be dated back to 0.04 to 3.46 MY ago. Due to the unaltered assembly and scaffolding, simulated genomes were assumed to have the same level of continuity compared to the original genome.

### Other genome metrics

Total scaffold size, scaffold N50, and contig N50 of a given genome assembly were calculated using the Perl script ‘assemblathon_stats.pl’ from Bradnam *et al.* (2013) (17) with parameter ‘*n* = 25’ that splits scaffolds into contigs when sequencing gaps reached 25 bp. Haploid genome size (1*n*) or C-value of the studied species were obtained from the Plant DNA C-values Database (release 6.0) (http://data.kew.org/cvalues/) (18) with manual curations using values from published genome studies.

The gene space completeness of genome assemblies was assessed by two pipelines, namely Core Eukaryotic Genes Mapping Approach (CEGMA v2.5) (19) and Benchmarking Universal Single-Copy Orthologs (BUSCO v3) (2). In CEGMA, a collection of 248 most conserved eukaryotic genes was searched against genome assembly with default parameters. In BUSCO, a set of 1,440 plant-specific orthologous genes, namely Embryophyta odb9, was used to search against genome assembly with parameters ‘–lineage_path embryophyta_odb9 –mode geno’. The completeness of gene space in a given genome assembly was defined by the proportion of completely matched proteins out of 248 conserved eukaryotic genes or 1,440 embryophyta genes.

### Calculation of LTR Assembly Index (LAI)

There are four steps to calculate LAI for a genome assembly: (i) obtain LTR retrotransposon candidates; (ii) retain all intact LTR-RTs by filtering out false candidates; (iii) whole-genome LTR-RT annotation; (iv) calculate LAI. In this study, LTR-RT candidates were obtained using LTRharvest (11) with parameters ‘-minlenltr 100 -maxlenltr 7000 -mintsd 4 -maxtsd 6 -motif TGCA -motifmis 1 -similar 85 -vic 10 -seed 20 -seqids yes’ and LTR_FINDER (10) with parameters ‘-D 15000 -d 1000 -L 7000 -l 100 -p 20 -C -M 0.85’. Both of these parameter sets were requiring minimum and maximum LTR length of 100 bp and 7 Kb, respectively, with at least 85% identity between two LTR regions of a candidate. High-confidence LTR retrotransposons with perfect micro-structures of terminal motifs and target site duplication (the ‘pass’ category) were identified from LTR-RT candidates using LTR_retriever (4) with default parameters, which were regarded as intact LTR retrotransposons. All possible LTR sequences in a given genome were annotated by RepeatMasker using the non-redundant LTR-RT library constructed by LTR_retriever and with parameters ‘-e ncbi -q -no_is -norna -nolow -div 40 -cutoff 225’ (Supplementary Figure S2). Estimation of raw LAI was performed using the equation Raw LAI = (Intact LTR element length / Total LTR sequence length) * 100, which was carried out by the LAI program deployed in the LTR_retriever package with window size set to 3 Mb and sliding step set to 300 Kb (‘-window 3000000 -step 300000’). The whole-genome raw LAI score is also generated in this procedure.

Since the raw LAI score is correlated with the activities of LTR-RT (see Results), such as LTR-RT amplification and removal, the mean identity of LTR sequences of the monoploid (1×) genome was used to correct these effects. To estimate the mean identity of LTRs, genomic sequences annotated as LTR regions were extracted and subjected to all-versus-all BLAST. The identity of each sequence hit that has the highest query coverage (except self-alignment) was used to estimate the whole-genome LTR identity. The correction factor of 2.8138 estimated using 20 high-quality long-read genomes was used to correct raw LAI scores with the equation LAI = raw LAI + 2.8138 × (94 – whole genome LTR identity). The LAI is set to 0 when raw LAI = 0 or the adjustment produces a negative value. Estimation of LTR identity and correction of raw LAI were also carried out by the LAI program. The mean age of intact LTR-RTs estimated by LTR_retriever was also used as an indicator of LTR-RT activity, but the age could be overestimated in draft genomes since young LTR-RTs are among the most poorly assembled. Although LAI is independent of total LTR-RT content, estimation of LAI is empirically not accurate when total LTR-RT content is less than 5% and intact LTR-RT content is less than 0.1%. To control for abnormally high LAI score, the regional LAI is down-scaled to 10% of the original score when total LTR-RT content is less than 1% in both whole-genome and regional LAI estimations. LAI is a default output of LTR_retriever since version 1.5 and freely available through GitHub under the GNU General Public License v3.0 (https://github.com/oushujun/LTR_retriever).

### Estimation of regional LAI

The PacBio long-read sequenced *O. sativa* cv. R498 rice genome was used to test four methods for regional LAI estimation. The genome was split into 5-Mb non-overlapping regions, which were treated independently for detection of intact LTR-RTs. A total of 72 regions were obtained after removing chromosome ends that were shorter than 5 Mb. Either the whole-genome LTR-RT library or the regional LTR-RT library (generated based on intact LTR-RTs in that region) were used to annotate total LTR-RTs in each region. Either the whole-genome mean LTR identity or the regional mean LTR identity was used to adjust for regional raw LAI. The whole-genome LAI was used to serve as the reference, which is slightly lower than the LAI that were calculated based on the standardized total LTR-RT content and LTR identity using the Nipponbare genome.

### Identification of low-quality candidate regions in the rice genome

LAI scores were calculated based on 3-Mb windows with 300-Kb steps and adjusted using the mean LTR identity on the rice reference genome (MSUv7). A cutoff value of 10 is used to identify low-quality candidate regions. The rice centromeres were identified by Cheng *et al.* based on the presence of the 155-bp CentO satellite repeat and the rice centromere-specific retrotransposon (http://rice.plantbiology.msu.edu) (20). Centromeric regions were defined based on the coordinate of centromeres with 1 Mb extended on both upstream and downstream regions. Sequence gaps were identified where the ambiguous character ‘N’ is presented in the genome sequence with the gap size equal or larger than 10 bp.

### Identification of solo LTRs

Solo LTRs were identified based on the whole-genome LTR-RT annotation generated by RepeatMasker (http://www.repeatmasker.org/). The non-redundant LTR library generated by LTR_retriever was used as the custom LTR-RT library for RepeatMasker. Annotation entries with Smith-Waterman scores < 300 and alignment lengths < 100 bp are removed for uncertainty. A sequence region is termed solo LTR if (i) it is annotated by an LTR region without any internal regions located within 300 bp adjacent to the target region; (ii) no nearby (the adjacent four annotation entries) sequence regions were annotated by the same LTR-RT entry and (iii) the length of the alignment hit accounts for at least 80% of the length of the solo LTR candidate. The script ‘solo_finder.pl’ for solo LTR identification is also included in the LTR_retriever program package. Percentage of solo LTR is calculated using the length of solo LTR over the length of all LTR-RT sequences.

### Statistical analyses

Fitting of linear models and test of significance (*F* test) were performed using the lm() function in R. Multiple comparisons were performed using R with Bonferroni correction. Manhattan plots were generated using the qqman package in R (https://github.com/stephenturner/qqman).

## RESULTS

### Construction of the LTR Assembly Index

Here we propose the LTR Assembly Index (LAI) for assessing the *de novo* assembly quality of intergenic and repetitive sequences. The LAI is a standardized metric based on LTR retrotransposons that account for the largest genome component in most plant genomes. The definition of raw LAI is described as follows:

\begin{equation*}{\rm{Raw}}\,{\rm{LAI}} = \frac{{{\rm{Intact}}\,{\rm{LTR}}\,{\rm{retrotransposon}}\,{\rm{length}}}}{{{\rm{Total}}\,{\rm{LTR}}\,{\rm{sequence}}\,{\rm{length}}}}\times 100\end{equation*}

Standardization of LAI is involved in detection of the total LTR-RT content in the genome and identifying high-quality intact LTR elements (see Materials and Methods for details). Intact LTR-RTs are identified by LTR_retriever (4), which recognizes a number of sequence features such as the complete long terminal repeat (LTR), di-nucleotide termini flanking the LTR region (usually 5′-TG..CA-3′), 4–6 bp target site duplication flanking the element, and alignment of protein sequences in the internal region. For the estimation of the denominator (total LTR-RT length), the non-redundant LTR-RT library (exemplars) generated by LTR_retriever was used to search the genome by the homology-based RepeatMasker program, then the length of all annotated sequences in the genome was summed up as the denominator. In cases where the degradation of LTR retrotransposons left unrecognizable sequence fragments, this estimation may be difficult to ascertain. To identify all LTR sequences in the genome, we progressively increased the divergence threshold in homology searches using RepeatMasker. The raw LAI score stabilized when sequence divergence increased to 40% in LTR-RT annotations of both rice and Arabidopsis (Supplementary Figure S2). Thus, the divergence rate of 40% is used for the estimation of total LTR-RT content in this study.

The content of LTR-RTs in a genome is a complex interplay between LTR-RT amplification and removal, which could be very different among species. Since young elements are more likely to remain intact, it is conceivable that raw LAI is influenced by the dynamics of LTR-RTs. For example, if a species has recent LTR-RT amplification, then more intact LTR-RT is present in the genome, resulting in an increase of raw LAI. In contrast, if there is little LTR-RT amplification, or a large quantity of intact LTR-RTs has been eliminated from the genome, the raw LAI of the genome would be very low due to the dearth of intact LTR-RTs. To quantify the outcome of LTR-RT dynamics, one possible way is to estimate the mean age of intact LTR-RTs in a genome. However, intact LTR-RTs are usually young and highly identical to each other and are often the poorest assembled component in genomes. Thus, assembly of intact LTR-RT is biased to older elements with higher diversity and their mean age is prone to be overestimated in draft genomes. On the other hand, the presence of LTR regions in a genome also reflects the amplification and removal of LTR-RTs, which could be measured by the identity of LTR regions in each LTR family. Because of the shorter length and higher diversity, assembly of LTR regions is relatively robust to genome quality. Moreover, the identity of LTR regions in a family is a more comprehensive indicator for amplification because it collects information from both intact elements and solo LTRs. Thus, we used the LTR identity to represent the dynamics of LTR-RTs, which is estimated using all-versus-all BLAST among all LTR regions in a genome (see Materials and Methods for details).

**Figure 1.**
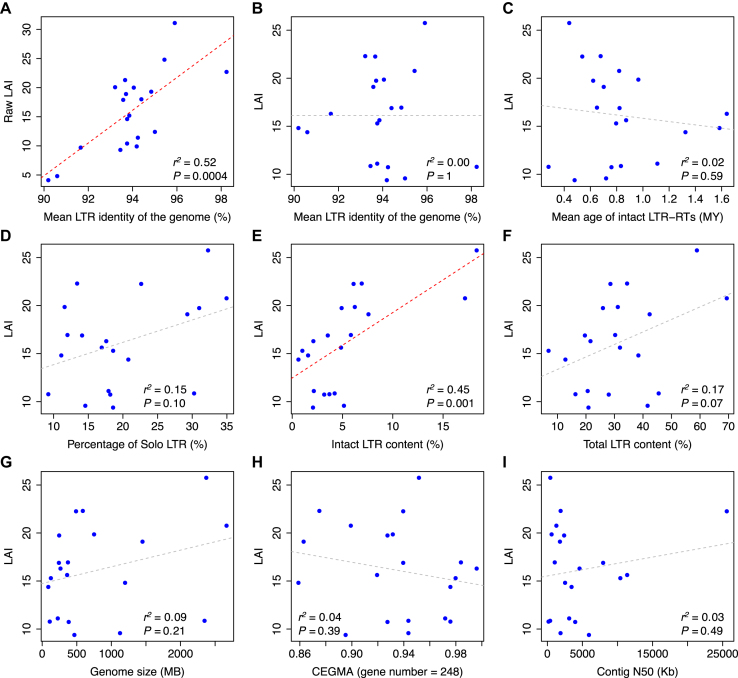
Characterization and correction of the LTR Assembly Index (LAI) using 20 high-quality plant genomes. (**A**) The raw LAI is linearly correlated with the mean LTR identity of the genome. The LAI adjusted based on the mean LTR identity is independent of (**B**) the mean LTR identity of the genome, (**C**) the mean age of intact LTR-RTs, (**D**) the percentage of solo LTR among all LTR sequences, (**F**) total LTR-RT content, (**G**) haploid genome size, (**H**) CEGMA gene set completeness, and (**I**) contig N50. (**E**) LAI is linearly correlated with intact LTR-RT content. Each blue dot represents one species. The coefficient of determination (*r*^2^) and *F-*test *P* value between x- and y-axis are indicated on each plot. Significant and non-significant linear regressions are indicated in red- and gray- dotted lines, respectively.

To test the relationship between raw LAI and the dynamics of LTR-RTs, we selected 20 plant genomes that were sequenced using long-read sequencing techniques and possess high quality as revealed by other genome metrics such as contig N50, BUSCO completeness, and CEGMA completeness (Supplementary Table S1; Materials and Methods). Our results show that the raw LAI score is linearly correlated with the mean LTR identity of these high-quality genomes (*r^2^* = 0.52, *P* = 0.0004, *F* test, which was used unless stated otherwise) (Figure 1A). We thus adjusted the raw LAI based on this relationship (Figure 1B), and the adjusted LAI becomes independent with the amplification time of LTR-RTs that is represented by the mean insertion time of individual intact LTR-RTs (*r*^2^ = 0.02, *P* = 0.59) (Figure 1C). There is no significant correlation between the adjusted LAI and solo LTR content that represents the removal of LTR-RT (*r*^2^ = 0.15, *P* = 0.10) (Figure 1D). These data indicate that adjusting the raw LAI using whole-genome LTR identity is effective. For simplicity, we use ‘LAI’ to replace the ‘adjusted LAI’ hereafter and in output of the LAI program.

To further study the age effect of LTR-RTs, we simulated three sets of genomes by artificially introducing different levels of random mutations to high-quality genome sequences of *Selaginella lepidophylla* (LAI = 11.1) (21), *Oropetium thomaeum* (LAI = 19.7) (22), and *Zea mays* chromosome 1 (LAI = 27.3) (13) as if they evolved 0.04–3.46 MY forward in time (Supplementary Figure S3). The LTR-RT evolution time of 3.46 MY approaches the detection limit of intact elements (Supplementary Figure S3), thus represents the most extreme case that the LAI program may encounter. As expected, the dramatic variation within raw LAI scores (∼30× difference between extremes) representing the effect of LTR amplification dynamics were almost eliminated by the correction using mean LTR identities of each simulated genome (∼1.4× difference between extremes) (Supplementary Figure S3), indicating that LAI is robust to LTR-RT insertion time.

### Characterization of the LTR Assembly Index

We characterized the relationship between LAI and other popular genome metrics using the high-quality genome dataset. As expected, LAI is linearly correlated with the content of intact LTR-RTs identified in these genomes (*r*^2^ = 0.45, *P* = 0.001) (Figure 1E). Moreover, no significant correlations were detected between LAI and total LTR-RT content (*r*^2^ = 0.17, *P* = 0.07) (Figure 1F), haploid genome size (*r*^2^ = 0.09, *P* = 0.21) (Figure 1G), total scaffold size (*r*^2^ = 0.14, *P* = 0.11) (Supplementary Figure S4C), CEGMA completeness (*r*^2^ = 0.04, *P* = 0.39) (Figure 1H), BUSCO completeness (*r*^2^ = 0.04, *P* = 0.37) (Supplementary Figure S4A), and contig N50 (*r*^2^ = 0.03, *P* = 0.49) (Figure 1I), suggesting that LAI is a new genome metric that is largely independent of existing quality metrics. In addition, a moderate correlation was observed between LAI and scaffold N50 (*r*^2^ = 0.22, *P* = 0.03) (Supplementary Figure S4B), indicating that high-quality scaffolding could improve the continuity of genome assemblies. In summary, LAI is robust among plant genomes with varying genome size, total LTR-RT content, and LTR-RT dynamics, indicating its potential in comparing assembly quality of different plant species.

**Figure 2.**
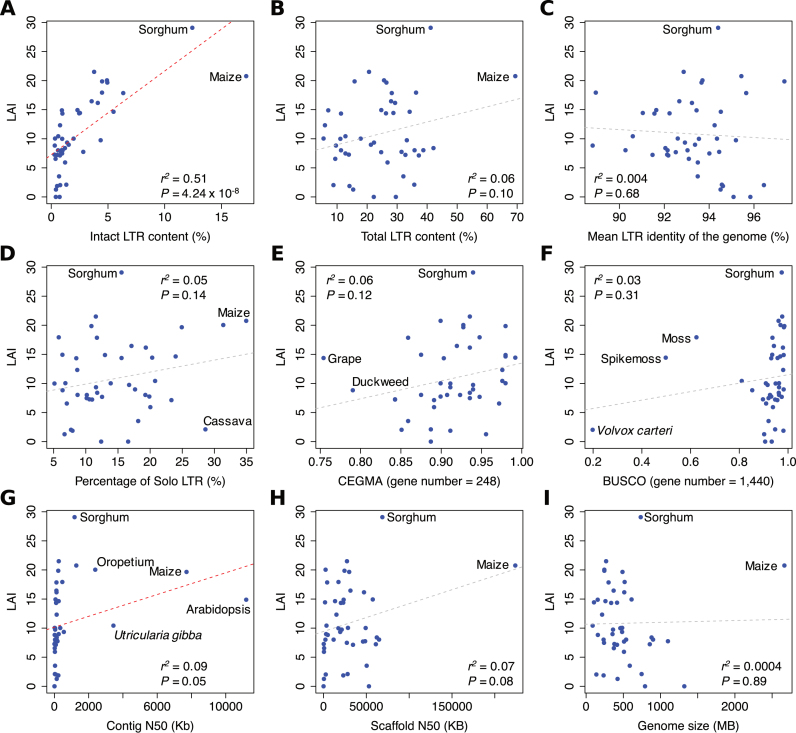
Relationships between LAI and other genome metrics among genomes with variable assembly quality. LAI is significantly correlated with (**A**) intact LTR-RT content (*r*^2^ = 0.51) and marginally correlated with (**G**) contig N50 (*r*^2^ = 0.09) and (**H**) scaffold N50 (*r*^2^ = 0.07). LAI is independent of (**B**) total LTR-RT content, (**C**) the mean LTR identity of the genome, (**D**) percentage of solo LTR among all LTR-RT sequences, (**E**) CEGMA completeness of 248 genes, (**F**) BUSCO completeness of 1,440 genes and (**I**) haploid genome size (*P* ≥ 0.10). Each dot represents a plant genome (*n* = 44) that contain > 5% of LTR sequence. The coefficient of determination (*r*^2^) and *F-*test *P* value between x- and y-axis are indicated on each plot with outliers also indicated. Significant and non-significant linear regressions are indicated in red- and gray-dotted lines, respectively.

To further test the performance of LAI, we utilized 44 publicly available plant genomes with varying quality, with most of them collected from Phytozome (Supplementary Table S1). Similar to the findings using high-quality assemblies, LAI is independent of total LTR-RT content (*r*^2^ = 0.06, *P* = 0.10) (Figure 2B) and genome size (*r*^2^ = 0.0004, *P* = 0.89) (Figure 2I), while linearly correlated with intact LTR-RT content (*r*^2^ = 0.51, *P* = 4.24 × 10^−8^) (Figure 2A) and marginally correlated with contig N50 (*r*^2^ = 0.09, *P* = 0.05) (Figure 2G) and scaffold N50 (*r*^2^ = 0.07, *P* = 0.08) (Figure 2H). Furthermore, there is no significant correlation between LAI and LTR dynamics represented by LTR identity (*r*^2^ = 0.004, *P* = 0.68) (Figure 2C) and solo LTR content (*r*^2^ = 0.05, *P* = 0.14) (Figure 2D). The BUSCO and CEGMA completeness are poor predictors of LAI (*r*^2^ ≤ 0.06, *P* ≥ 0.12) (Figure 2E and F), indicating that LAI is characterizing a sequence space different from the gene space. In contrast, the CEGMA and BUSCO evaluations are congruent with each other (Supplementary Figure S5), despite the fact that BUSCO relies on different gene sets for evaluation of specific organisms (i.e. alga, mosses, and lycophytes). Again, these results demonstrate that LAI is a new genome metric for evaluating the assembly of the intergenic and repetitive sequence space.

Among the 44 genomes we tested, the sorghum (*Sorghum bicolor*) genome shows the highest LAI (LAI = 29.1) and appears to be an outlier (Figure 2). We further examined the structural features of LTR elements in these genomes and found that the internal regions of LTR-RTs in sorghum is among the longest, which is 7.3 Kb comparing to 5.6 Kb of all genomes in average (Supplementary Table S1). Similarly, *Setaria viridis* also has very long internal regions (mean = 7.3 Kb) but poor LAI (LAI = 7.7) (Supplementary Table S1). Thus, the high LAI score of sorghum genome is likely attributed to a combination of high assembly quality with the presence of elements with long internal regions (see Discussion).

### Comparison of sequencing techniques using LAI

To compare the assembly continuity of new sequencing techniques to the gold standard, the bacterial artificial chromosome (BAC) technique, we collected high-quality BAC sequences from different plant species in NCBI. These sequence assemblies were manually curated by uploaders and serve as the gold standard for genome benchmarking. After screening for species with more than 3 Mb BAC sequences available (mean size: 54 Mb), 21 plant species with 14,826 high-quality BAC sequences were retained (see Materials and Methods for details). We also collected whole-genome sequences from 70 plant species that were sequenced by various techniques (Supplementary Table S1).

**Figure 3.**
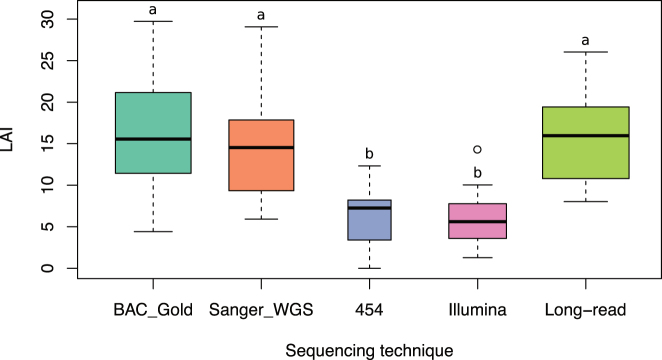
Comparison of LAI scores among genomes sequenced using different techniques. Genomic sequences of a total of 90 samples were collected from Phytozome, NCBI, and other sources (see Materials and Methods for details) and further placed into different categories based on their major sequencing techniques. BAC_Gold, fully sequenced BAC samples from 21 species with three are whole-genome sequenced (rice, maize, and Arabidopsis). 454, Roche 454 sequencing. Illumina, Illumina dye sequencing. Sanger_WGS, Sanger-based whole-genome shotgun sequencing. Long-read, long-read sequencing including PacBio sequencing (22 species) and Oxford Nanopore sequencing (two species). The width of each box represents the relative sample size. Black bars indicate the median value of each group. Different letters on each box indicate significantly different LAI values between categories (two-tailed *t*-test, Bonferroni adjustment).

As shown above, LAI is independent of genome size and total scaffold size (Figures 1G and 2I; Supplementary Figure S4C), the regional LAI (e.g. BAC LAI) is thus comparable to genomic LAI. However, the calculation of regional LAI could be biased by the LTR-RT library generated from only a fraction of the whole genome, especially for the estimation of total LTR-RT content and LTR identity (Supplementary Figure S6). To accurately estimate the regional LAI, we used the whole-genome LTR-RT library to annotate all LTR sequences in the focal region and the whole-genome LTR identity to adjust for the LTR-RT dynamics (see Materials and Methods for details). After the adjustment, the regional LAI could accurately reflect the quality of the whole genome (Supplementary Figure S6). We thus used this method to calculate the BAC LAI for comparison to other genomes. As expected, high-quality BAC sequences possess one of the highest LAI scores among existing techniques, with a mean LAI score of 15.5, which has been served as the gold standard for high-quality sequencing (Figure 3).

While NGS techniques (i.e. Illumina sequencing and Roche 454 sequencing) have massively reduced the cost of sequencing a new genome, their ability to resolve repetitive sequences is very limited (14,23). Thus, assemblies mainly based on short reads usually have LAI scores below 10 (5.9 in average) and among the lowest of all sequencing techniques (Figure 3). Even for the very compact Arabidopsis genome that only contains 21% of repetitive sequences including 7% LTR-RTs (4,24,25), assembling a continuous genome using NGS reads is still challenging. The Arabidopsis Nd1 strain sequenced by Pucker *et al.* using Illumina short reads (26), with the chromosome-level scaffolding, has an LAI score of 6.9. The Sanger whole-genome shotgun (WGS) technique featured with low-coverage (6-9×) Sanger sequencing also yielded high-quality genomes (LAI = 14.4 in average) (Figure 3). However, the cost-ineffective and labor-intensive nature made it difficult to construct high-coverage BAC libraries and close gaps, especially for large genomes. More recently, single-molecule long-read sequencing has become popular in the genome sequencing market. The GC-unbiased PacBio technique and the super-long length nanopore technique enable efficient resolution of complicated sequence structures (27,28). As a result, the repetitive and intergenic sequence in these long-read assembled genomes is the best assembled among different sequencing techniques (LAI = 15.7) (Figure 3). Although there is no statistical difference between LAI scores of Sanger WGS genomes and long-read genomes (Figure 3), the later technique tends to produce genomes with higher quality. For example, 19 out of 24 long-read genomes (79%) possess LAI scores higher than 10, while only 12 out of 18 Sanger WGS genomes (67%) fall in this category.

### Identification of low-quality genomic regions

**Figure 4.**
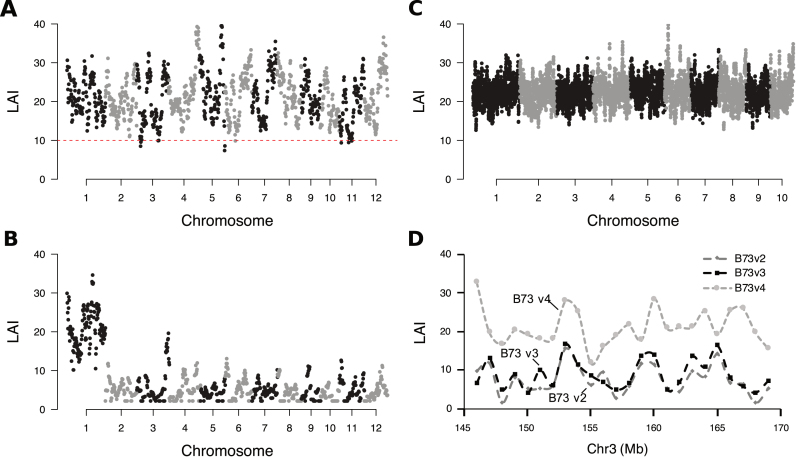
LAI score reveals regional assembly quality of repetitive sequences. LAI scores in genomic regions of (**A**) rice var. Nipponbare MSUv7, (**B**) rice var. Kasalath, (**C**) maize var. B73 v4, and (**D**) three versions of the maize B73 genome. X-axes indicate chromosomes of each genome. Each dot represents LAI score of a 3 Mb-sliding window with 300-Kb increment, which was adjusted by whole-genome LTR identity. (A) A genome-wide cutoff (LAI = 10) shown by the red-dotted line is used to identify low-continuity candidate regions for further improvement. (B) Chromosome 1 was assembled based on a BAC physical map by Kanamori *et al.* (29), while other chromosomes were constructed based on the mapping to the Nipponbare reference genome (30). (D) Example regions from the maize chromosome 3 show improvements in assembly quality over genome version updates.

As demonstrated above, LAI is independent of total LTR-RT content (Figures 1F and 2B) and genome size (Figures 1G and 2I; Supplementary Figure S4C). Therefore, with the accurate estimation of regional LAI (Supplementary Figure S6), our method can be applied to visualize the local assembly quality of a genome. For this purpose, we computed LAI scores of genome assemblies based on 3 Mb-sliding windows with 300-Kb increment. Results show that the maize reference genome (B73 v4) sequenced by PacBio long reads has very high LAI scores evenly distributed across the assembly (Figure 4C). Further visualization of LAI scores in three versions of the maize reference genome in a region of chromosome 3, for example, show successive gains in assembly quality (Figure 4D).

The use of different sequencing and assembly methods also affects the quality of sequences within a genome. For example, the genome assembly of the rice variety Kasalath shows exceptional quality of chromosome 1 (LAI = 20.9) (Figure 4B), which is sequenced using the Sanger WGS technique and assembled based on a BAC physical map (29,30). In contrast, other chromosomes in this assembly exhibit very low quality (LAI = 4.0) (Figure 4B), which were sequenced by short reads and constructed solely based on mapping short contigs to the reference genome (var. Nipponbare) (30).

Even the most completed genome contains draft regions. Using the window-based LAI, we identified seven such candidate regions in the rice reference genome (MSUv7) with LAI scores < 10 (Figure 4A), which contain 29% of the sequencing gaps and is significantly more than other regions with higher LAI scores (*P* < 0.0001, two-tailed chi-squared test) (Supplementary Table S2). Although LTR removal might be suppressed in pericentromeric regions with low recombination rate (8,24), testing on the rice chromosomes 4 and 8 with fully resolved centromeres (31,32) reveal no difference of LAI scores between centromeric regions and other chromosomal regions (*P* = 0.96, two-tailed *t*-test), indicating that the LAI is not significantly different between genic and pericentromeric regions.

### LAI reveals and facilitates genome improvement

**Figure 5.**
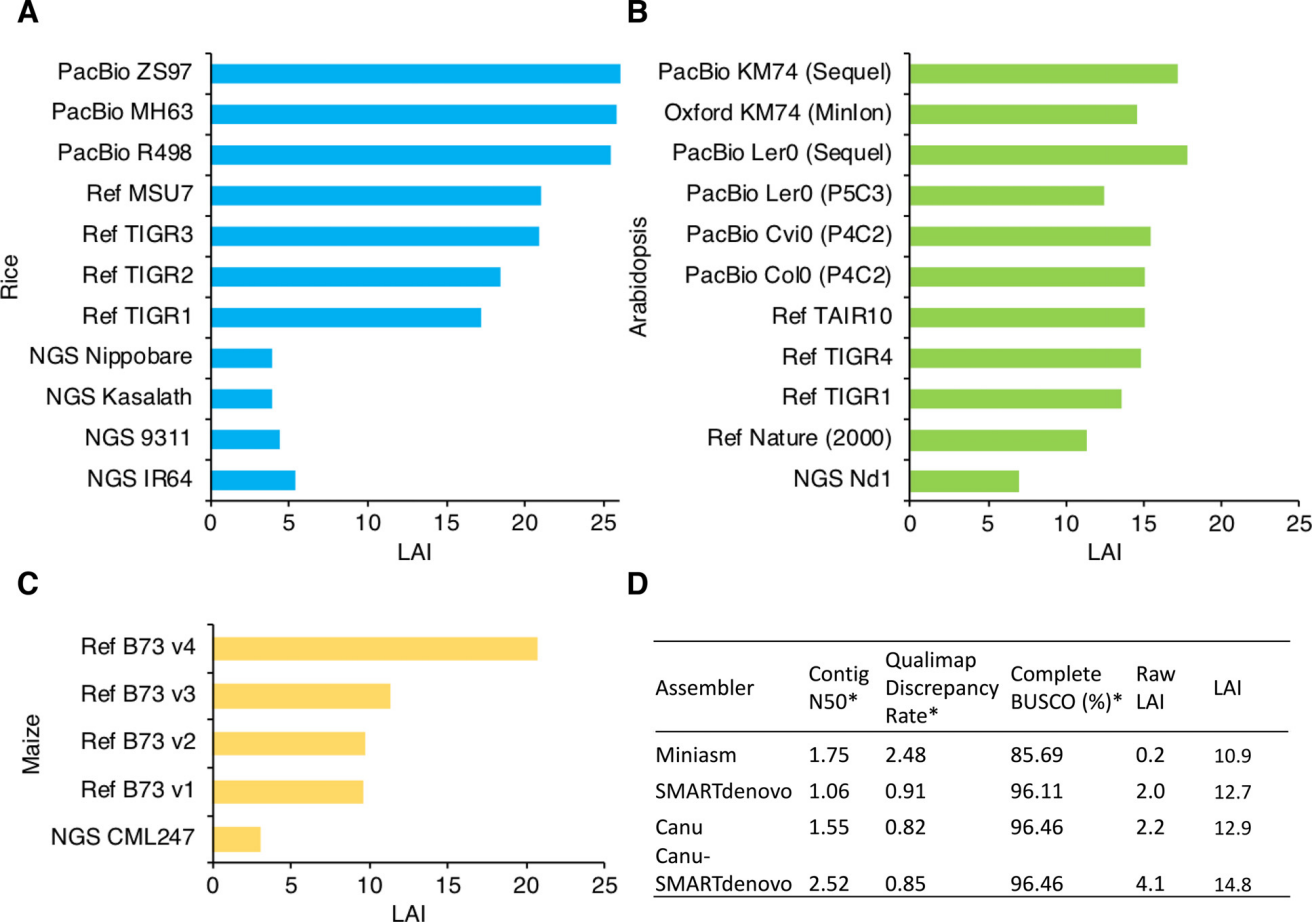
LTR Assembly Index of model plant genomes. LAI score of (**A**) rice genomes, (**B**) Arabidopsis genomes, and (**C**) maize genomes. Reference genomes were labeled as Ref with version number indicated. All reference genomes were sequenced by the Sanger-based BAC-by-BAC approach, with the exception of the maize genome Ref B73 v4, which was generated through the PacBio long-read sequencing technique. NGS, next-generation sequencing. PacBio, PacBio long-read sequencing. Oxford, Oxford Nanopore long-read sequencing. (D) Four versions of the *Solanum pennellii* genome assembled using different assemblers with the same batch of Oxford Nanopore sequencing data. *Data adapted from Schmidt *et al.*(16).

To assess the improvement of genome sequencing and assembly over time, we computed and compared the LAI score of model plant genomes with multiple assembly updates available. The results show that genomes sequenced using the Sanger-based technique and BAC-based scaffolding are associated with very high LAI scores, ranging from 10 to 21 (Figure 5). However, the improvement of Sanger-based genomes over time was marginal in regard to the quality of intergenic and repetitive sequences (Figure 5; Supplementary Figures S7 and S8). As we observed above, NGS-based genomes possess very low LAI scores (Figure 3). Similarly, the NGS versions of these model species also possess very low sequence continuity (Figure 5). In many cases, the long-read technique yielded high-quality assemblies that surpass the quality of reference genomes (Figure 5), indicating a promising future of genome sequencing.

Using the LAI program, it is possible to distinguish different assemblers and probably assembly parameters. For the genome of *Solanum pennellii* sequenced using the Oxford Nanopore long-read technique, sequencing reads were assembled using four different approaches which all yielded comparable quality as revealed by contig N50, mapping discrepancy, and BUSCO completeness (16). By introducing the tie-breaking LAI, we revealed that the Canu-SMARTdenovo approach produced a much higher sequence continuity, agreeing with the highest contig N50, BUSCO completeness, and the second lowest mapping discrepancy of the assembly (Figure 5D).

## DISCUSSION

As LTR-RT sequences challenge the current sequencing technique and assembly algorithms, the assembly quality of these sequences, in turn, could reflect the quality of the whole genome assembly. Intact LTR-RTs serve as a sensitive indicator due to the difficulties in resolving its complete structure. It would be optimal if all intact LTR-RTs of a genome are known, so that the genome quality could be evaluated by the percentage of fully assembled LTR-RTs. However, measuring the exact amount of intact LTR-RT is impossible until the assembly reaches its perfection, which is the dilemma of evaluating genome quality solely based on intact LTR-RTs. Without knowing the content of intact LTR-RTs, one possible way is to control for the factors that alternate the level of intact LTR-RTs between genomes, such as the total LTR-RT content (including intact and fragmented LTR-RTs) and the activity of LTR-RTs (including the amplification and removal of LTR-RTs). After reconciling the radical differences of these two factors between genomes, we developed LAI for interspecific comparisons of assembly continuity.

In this study, we demonstrate that LAI is a universal metric that is robust to genome size (Figures 1G and 2I; Supplementary Figure S4C) for the evaluation of repetitive and intergenic sequence space. It is worth noting that the calculation of LAI relies on the identification of intact LTR-RTs to estimate the total LTR-RT content. After adjustment, although LAI is insensitive to LTR-RT content (Figures 1F and 2B) and LTR-RT dynamics (Figures 1B–D and 2C–D) in general, for genomes with limited LTR sequence (intact LTR-RT < 0.1%, total LTR-RT < 5%), the number of detectable intact elements would be insufficient to cover all LTR-RT related sequences for accurate estimation. This is the case for many non-plant species such as human (*Homo sapiens*, intact LTR-RT 0.02%), zebrafish (*Danio rerio*, total LTR-RT 3.3% (33)), and nematode (*Caenorhabditis elegans*, total LTR-RT 0.4% (34)). For the identification of intact LTR-RT, many existing methods are available, such as LTR_STRUC (35), LTR_FINDER (10), LTRharvest (11), and LTR_retriever (4). However, based on our previous study, only LTR_retriever possesses a low level of false discovery rate and effectively eliminates misassembled LTR elements (4). Thus, the LAI score estimated based on LTR_retriever not only indicates the amount of LTR-RT sequences that are assembled, but also reflects the correctness of assembly.

Widely recognized as one of the best-sequenced plant genomes, the Arabidopsis reference genome has a lower LAI score (LAI = 14.9) compared to the reference genomes of rice (LAI = 21.1) and maize (LAI = 20.7), which could be due to the low abundance of LTR sequence (7%) and the existence of unclosed sequencing gaps (36). Even the reference genomes of rice and Arabidopsis sequenced using the BAC-by-BAC approach and regarded as the ‘gold standard’ for eukaryotic genomics, their genome assemblies still contain many gaps, misassemblies, and missing significant amounts of sequences which are mainly comprised of rDNA and centromeric sequences (36,37). For example, only two short rice centromeres (on chromosomes 4 and 8) were fully sequenced to date (31,32), while the rests are still infused with physical gaps or in draft stage (Supplementary Table S2) (38). Benefiting from the long-read-length nature, long-read techniques are able to span larger regions that are enriched with nested transposon insertions and highly identical repeats, including some of the relatively short centromeric regions (36,37), which yields higher LAI scores comparing to those of reference genomes (Figure 5). However, many centromeres and rDNA arrays span several Mb and have near 100% identity in these genomes, which are still unresolved even using the ‘state-of-the-art’ long-read techniques and remain the major challenge for eukaryotic genomics (36).

For comparison between assemblies of the same species, it is recommended to use the raw LAI score because LTR-RT dynamics is comparable within species (Figure 5D). Furthermore, raw LAI is computationally more efficient than LAI, because the former does not require the calculation of the genome-wide LTR identity. Thus, raw LAI could be helpful for users to quickly select a high-quality genome with multiple available versions or for genome researchers to iteratively improve the genome assembly by selecting assemblers and parameters that yield the highest raw LAI. After adjusting for LTR dynamics using LTR identity of the genome, the LAI becomes robust for interspecific comparison (Figures 1–3; Supplementary Figures S3 and S4). For example, we identified the long-read-based *Utricularia gibba* genome, the smallest flowering plant genome being sequenced so far (101 Mb) (39), has high-quality gene space and contiguity given the CEGMA completeness of 0.98 and contig N50 of 3.4 Mb. However, the *U. gibba* genome has extraordinary low LAI score (raw LAI = 4.8) due to very limited retrotransposition activities in the past few million years (40). After adjusting for LTR dynamics, the LAI score recovers to 14.4, which agrees with the completeness of gene space and overall high quality, indicating that the adjustment is effective.

Our data indicate that the BAC-by-BAC approach still serves as the ‘gold standard’ for genome sequencing (Figure 3). However, due to its high cost, it is unrealistic for the BAC-based technique to dominate whole-genome sequencing in future. Alternatively, a small number of fully sequenced BACs can be used to quantify sequencing and assembly errors in a genome generated by other techniques. Different from this approach, the LAI program studies the sequence contiguity purely based on the genome assembly itself without further input, which could significantly ease the evaluation procedure and provide a generic result for readers and genomic researchers about the continuity of the focal genome assembly. Theoretically, LAI score could range from 0 to 100. However, by comparing to sequences generated through the ‘gold standard’ BAC-by-BAC approach, LAI score of greater than or equal to 20 indicates high quality. In this regard, we further propose a genome classification system for the assembly of repetitive and intergenic sequence space using LAI: draft quality, with LAI score less than 10; reference quality, with LAI score ranges from 10 to 20; and gold quality, with LAI score greater than 20 (Table 1).

**Table 1.** Classification of repetitive sequence assemblies using the LTR Assembly Index (LAI)

Category	LAI	Examples
Draft	0 ≤ LAI < 10	Apple (v1.0), Cacao (v1.0)
Reference	10 ≤ LAI < 20	Arabidopsis (TAIR10), Grape (12X)
Gold	20 ≤ LAI	Rice (MSUv7), Maize (B73 v4)

Among the genomes we have analyzed, the sorghum genome receives the highest LAI score (LAI > 29) (Supplementary Table S1), which is somewhat surprising. However, scaffold N50 and contig N50 of the sorghum genome is among the highest (Figure 2G and H), which could be attributed to the use of Sanger sequencing followed by manual curation (41) (Supplementary Figure S8). Besides, the assembly of the sorghum genome was facilitated by a physical map, which has been shown very powerful for construction of a continuous assembly (Figure 4B). In regard to the internal region size of LTR elements, the sorghum genome is one of the largest (Supplemental Table S1). Longer internal regions would lead to an increased size of intact elements, resulting in an increased LAI value. Nevertheless, it is clear that long internal region does not guarantee a high LAI score in that the LAI of the *Setaria viridis* genome is only 7.7, which is consistent with its short contig N50 (68 Kb). It is conceivable that when the contig is short, long LTR elements are less likely to be assembled, resulting in low LAI. Together, the high LAI value of the sorghum genome is due to both its high quality and long internal regions of LTR elements.

Using LAI, genome researchers can now evaluate the quality of their genome assembly, compare assembly quality between different versions, select for the best-performed assembler, and acquire a perception of the quality of their genomes by comparing to other species. This is the first time the continuity of intergenic and repetitive sequence assembly can be quantified and compared across species. With the fast development of sequencing techniques and assemble algorithms, genome sequencing itself is shifting from being the major focus of a study to serving as the foundation to answer more biological questions that cover a broad, if not all, fields of biological research. In this regard, LAI is a basic yet important quality check for genome assemblies.

## DATA AVAILABILITY

Scripts and user's guide for computing LAI scores were included in the latest release of the open source package LTR_retriever in GitHub: https://github.com/oushujun/LTR_retriever.

## Supplementary Material

Supplementary Data
