# Supplementary material for: Assessing genome assembly quality using the LTR Assembly Index (LAI)
Source: Nucleic Acids Res. 2018 Aug 10;46(21):e126. doi: 10.1093/nar/gky730 (PMC6265445; doi:10.1093/nar/gky730)
Supplement: Supplementary Data [file gky730_supplemental_files.zip › Supplementary data.pdf]

Assessing genome assembly quality using the LTR Assembly Index (LAI)

Ou, Chen, and Jiang (2018)

SUPPLEMENTARY FIGURES AND TABLE

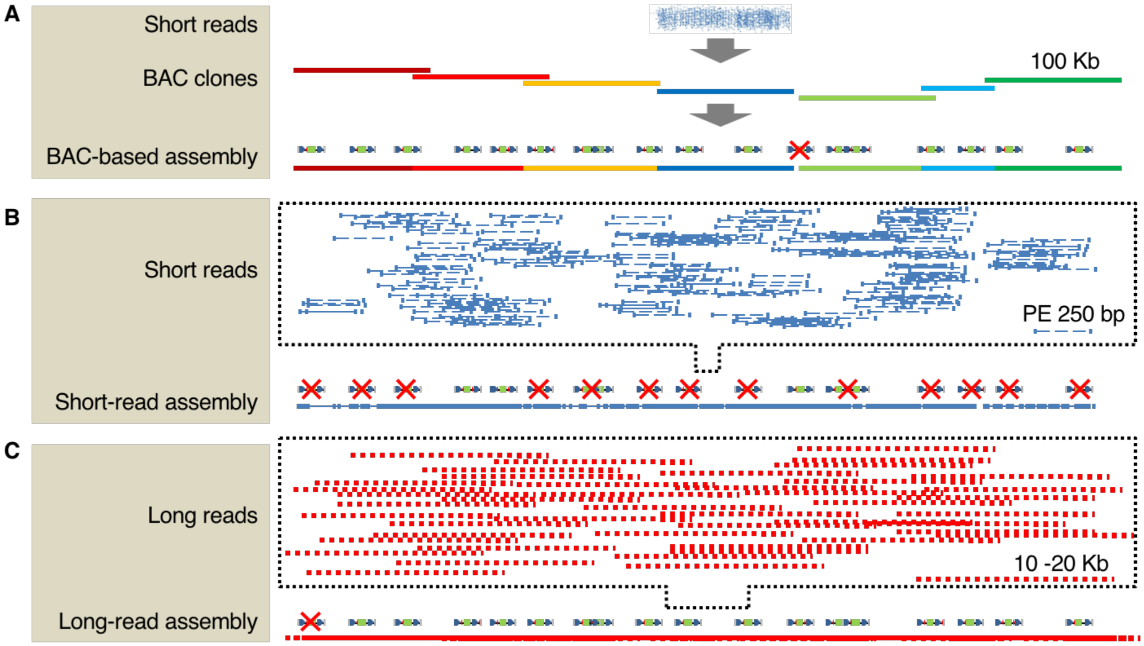

Supplementary Figure S1. Illustration of three genome sequencing schemes on a 1 Mb generic genome (1n = 1x = 1) and demonstration of the assembly quality reflected by the LTR Assembly Index. (**A**) Bacterial artificial chromosome (BAC)-based genome assembly. The genome is fragmentized and stored in BACs (~100 Kb in length) and each BAC is sequenced separately. BACs are assembled into the pseudochromosome level based on minimum tiling path or a physical map. (**B**) Short-read-based next-generation sequencing. The genome is shuffled into random short DNAs (~1.5 Kb in length) and each read is sequenced on both ends for about 250 bp by polymerase chain reactions (PCR). Sequenced reads are aggregated into contigs using computer algorithms and assembled into the scaffold level based on physical or optical maps. (**C**) Long-read-based next-generation sequencing. The genome is shuffled into random long DNAs (~15 Kb in length), and each read is sequenced based on single-molecule sequencing techniques. Sequencing errors of each read are corrected using consensus algorithms and assembled into the pseudochromosome level based on physical or optical maps. The whole genome length is ~1 Mb with a number of detectable intact LTR retrotransposons (LTR-RTs) labeled on top of chromosome assemblies. LTR-RTs that are not recognizable by structure-based methods due to sequencing defects were crossed out. Fragmented LTR-RTs and LTR sequence remnants are not labeled. The length between different components of the figure is roughly scaled to size.

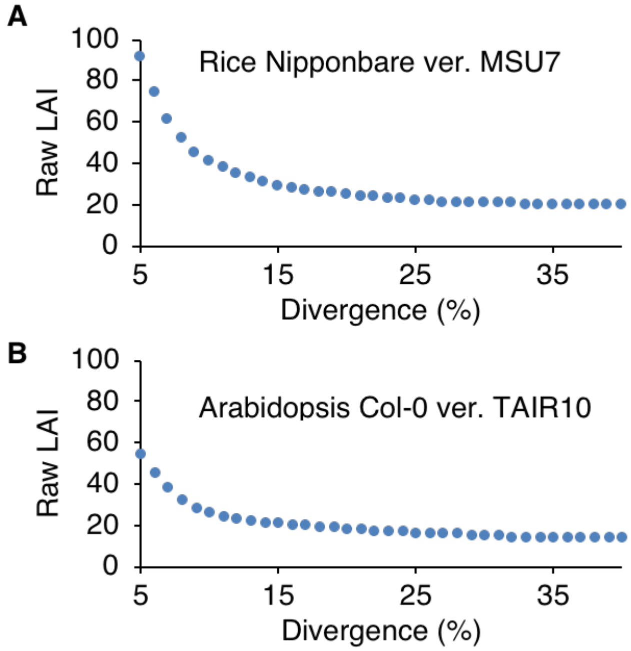

Supplementary Figure S2. The raw LAI score stabilized when sequence divergence for homology search increased to 40%. Raw LAI scores of (**A**) the rice reference genome (*Oryza sativa* ssp. *japonica* cv. Nipponbare ver. MSU7) and (**B**) the Arabidopsis reference genome (*Arabidopsis thaliana* cv. Columbia-0 ver. TAIR10) based on RepeatMasker annotation divergence from 5% to 40%.

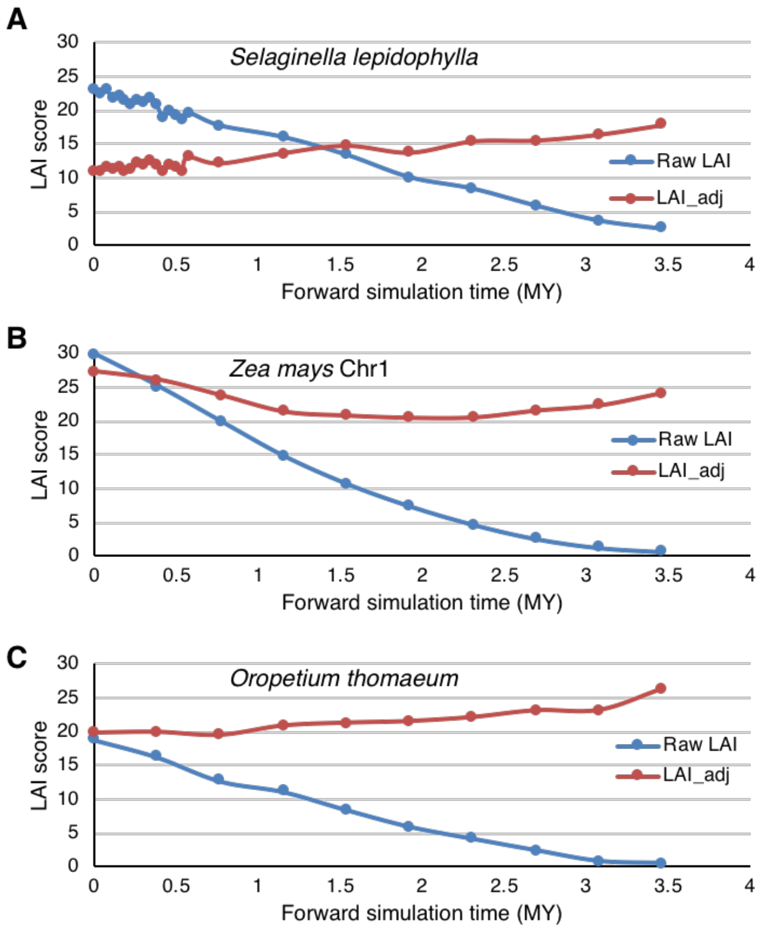

Supplementary Figure S3. LAI is robust to LTR-RT amplification time after adjustment. The reliably sequenced genome sequences of *Selaginella lepidophylla* (**A**), *Zea mays* chromosome 1 (**B**), and *Oropetium thomaeum* (**C**) were used for the simulation study. To simulate genome evolutions forward in time, random mutations from 0.1% to 9% equivalent to evolution times of 0.04 to 3.46 MY (x-axes; $\mu = 1.3 \times 10^{-8}$ per bp per year) were introduced to the original genome sequence (x = 0). Each dot represents a simulated genome with the raw LAI shown in blue and the adjusted LAI shown in red. The adjustment of LAI scores was based on the mean identity of LTR regions in each simulated genome.

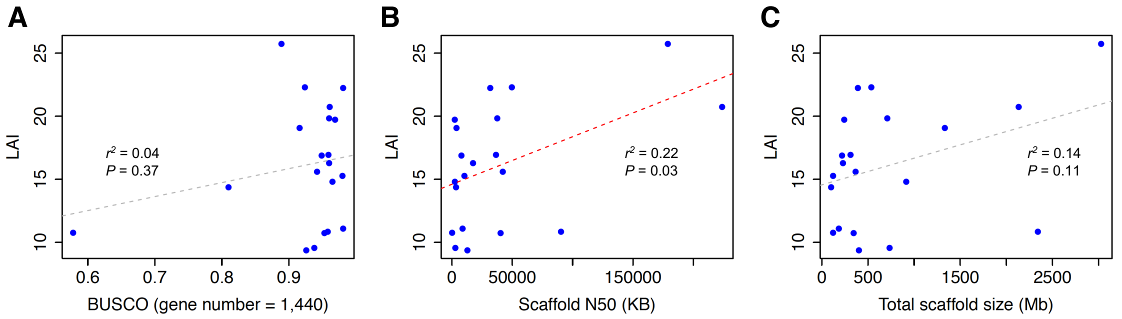

Supplementary Figure S4. LAI is not correlated with BUSCO (A) and total scaffold size (C), and correlated with scaffold N50 (B) in 20 high-quality genomes. Raw LAI scores were adjusted using the mean LTR identity of each genome. High-quality long-read genomes were selected based on BUSCO and CEGMA completeness, and contig N50 (see **Materials and Methods** for details). Each blue dot represents one species. Coefficient of determination (r^2) and *F*-test *P* value between x- and y-axis are indicated on each plot. Significant and non-significant linear regressions are indicated in red- and grey- dotted lines, respectively.

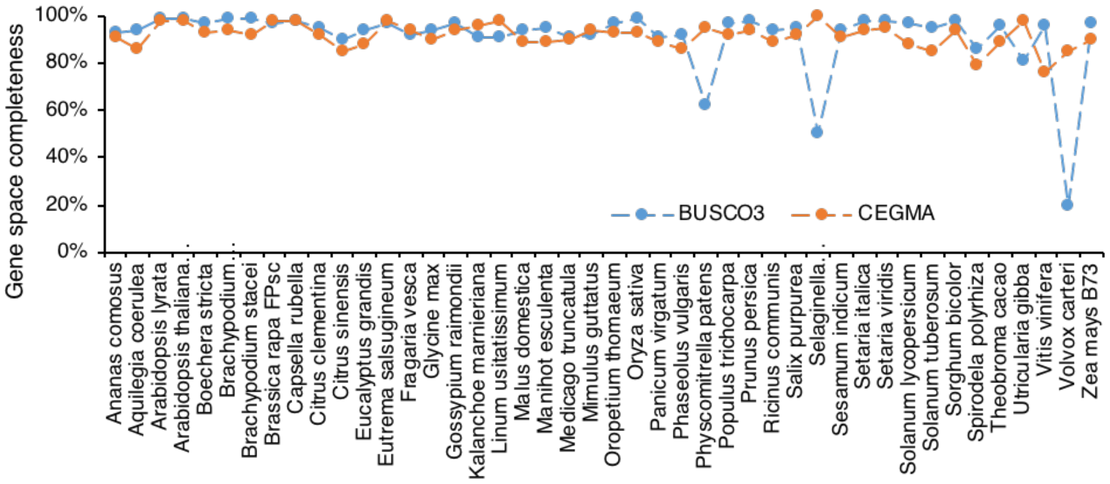

Supplementary Figure S5. BUSCO3 (gene number = 1,440) and CEGMA (gene number = 248) methods yield similar results in assessing gene space completeness. Species names of 44 plant genomes are indicated in the x-axis.

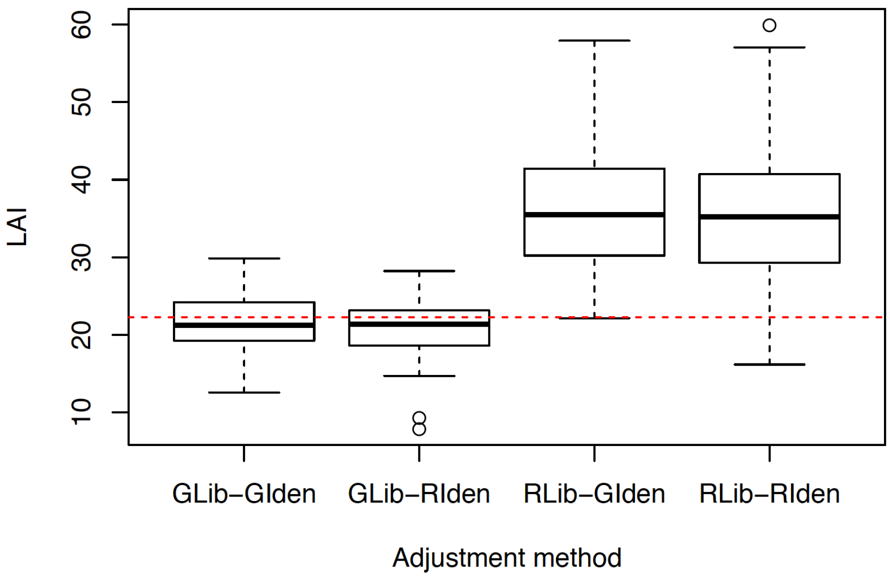

Supplementary Figure S6. Comparison of four adjustment methods for the estimation of regional LAI. The *Oryza sativa* cv. R498 genome was split into 5 Mb non-overlapping regions, which were treated independently for detection of intact LTR-RTs. Regional LAI scores were estimated based on four methods as indicated on the x-axis. GLib, total LTR-RT content of each region was annotated by the whole-genome LTR-RT library. RLib, total LTR-RT content of each region was annotated by the LTR-RT library generated from that region. GIden, the whole-genome LTR identity was used for raw LAI adjustment. RIden, the regional LTR identity was used for raw LAI adjustment. The whole-genome LAI = 22.3 is indicated by the red-dotted line.

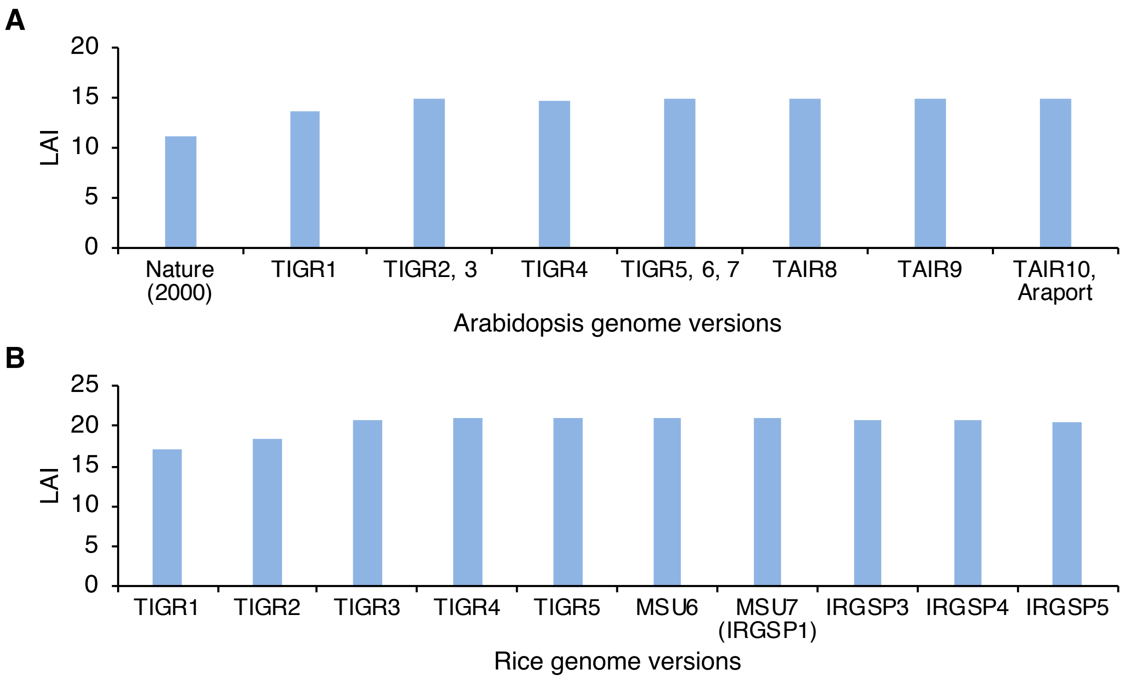

Supplementary Figure S7. LTR assembly score (LAI) of legacy versions of the Arabidopsis genome (A) and the rice genome (B). X-axes represent different genome assembly with version number indicated. TIGR, The Institute for Genomic Research; TAIR, The Arabidopsis Information Resource; MSU, Michigan State University; Araport, the Arabidopsis Information Portal; IRGSP, the International Rice Genome Sequencing Project. The rice genome versions IRGSP3-5 are predecessors of the MSU7/IRGSP1 version.

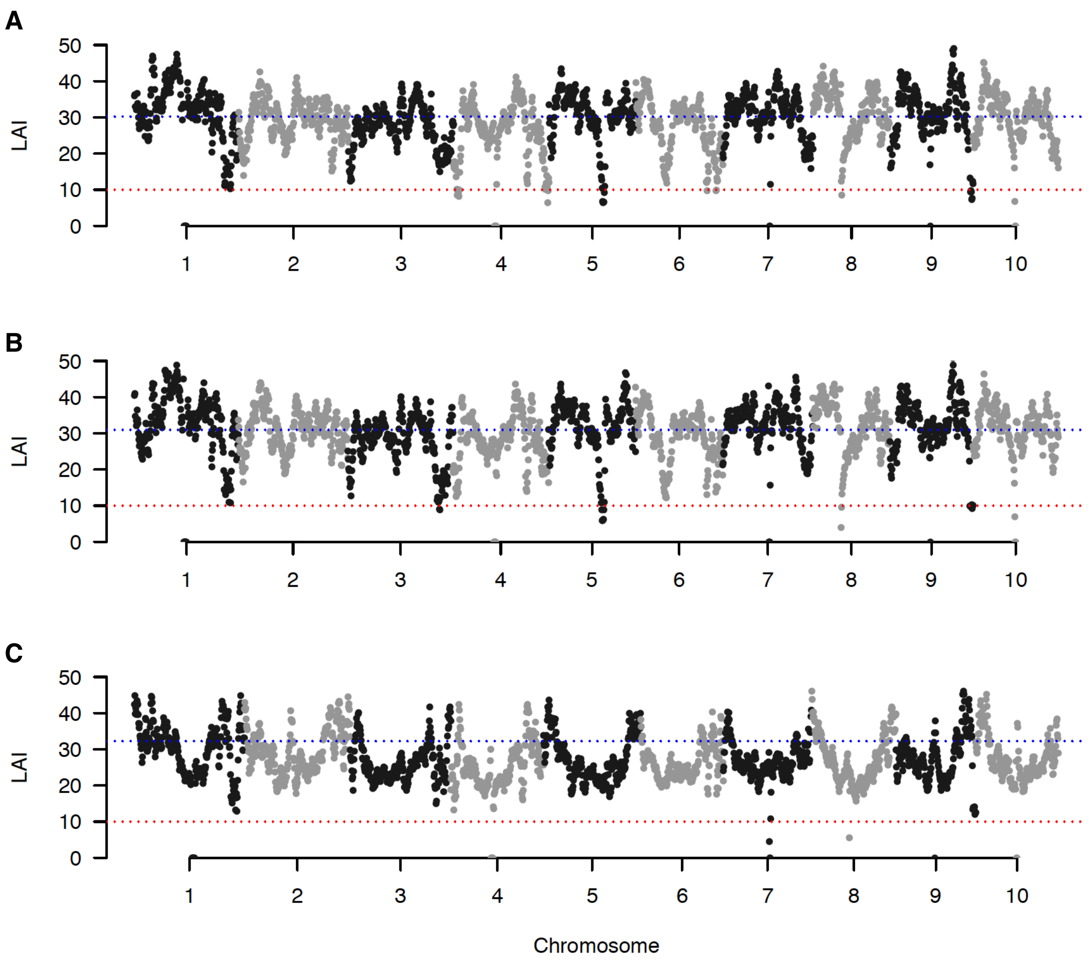

Supplementary Figure S8. LAI score distribution of three versions of the sorghum genome. (**A**) The v1.0 assembly sequenced using the Sanger whole-genome shotgun technique. Contig N50 = 195 Kb. (**B**) The v2.0 assembly improved by incorporation of 211 Mb of finished genic sequence. Contig N50 = 420 Kb. (**C**) The NCBIv3 version with extensive improvement. Contig N50 = 1.31 Mb. X-axes indicate chromosomes of each genome. Each dot represents LAI score of a 3-Mb sliding window with 300 Kb per step, which was adjusted by LTR identity of the genome. Red-dotted lines indicate LAI = 10. Blue-dotted lines indicate whole-genome LAI of each assembly.

Supplementary Table S2. Regions in the rice reference genome (MSU7) with adjusted LAI scores less than 10

Chromosome	Start	End	Intact LTR-RT	Total LTR-RT	LAI	Centromeric	# of gaps	Gap Length (bp)	% of solo LTR*
1-12	1	373245519	4.97%	26.36%	21.10	Yes	240	115885	25.00%
3	4200001	7500000	0.80%	11.56%	9.04	No	1	1000	22.68%
3	22800001	26400000	1.87%	23.97%	9.95	No	0	0	19.82%
5	26700001	29958434	0.91%	15.32%	7.99	No	7	2500	22.36%
6	11100001	14100000	2.30%	29.90%	9.88	No	0	0	23.98%
11	1800001	4800000	1.25%	17.38%	9.38	No	0	0	23.30%
11	9600001	12600000	2.99%	41.04%	9.46	Yes	61	7000	22.98%
11	12900001	15900000	3.49%	45.24%	9.88	Yes	1	1000	26.02%

*The percentage of total LTR-RT contributed by solo LTRs.
